# Resting-state connectivity in neurodegenerative disorders: Is there potential for an imaging biomarker?

**DOI:** 10.1016/j.nicl.2018.03.013

**Published:** 2018-03-16

**Authors:** Christian Hohenfeld, Cornelius J. Werner, Kathrin Reetz

**Affiliations:** a RWTH Aachen University, Department of Neurology, Aachen, Germany; b RWTH Aachen University, Section Interdisciplinary Geriatrics, Aachen, Germany; c JARA-BRAIN Institute Molecular Neuroscience and Neuroimaging, Forschungszentrum Jülich GmbH and RWTH Aachen University, Aachen, Germany

**Keywords:** Neurodegeneration, Resting-state, fMRI, Review, Biomarker

## Abstract

Biomarkers in whichever modality are tremendously important in diagnosing of disease, tracking disease progression and clinical trials. This applies in particular for disorders with a long disease course including pre-symptomatic stages, in which only subtle signs of clinical progression can be observed. Magnetic resonance imaging (MRI) biomarkers hold particular promise due to their relative ease of use, cost-effectiveness and non-invasivity. Studies measuring resting-state functional MR connectivity have become increasingly common during recent years and are well established in neuroscience and related fields. Its increasing application does of course also include clinical settings and therein neurodegenerative diseases. In the present review, we critically summarise the state of the literature on resting-state functional connectivity as measured with functional MRI in neurodegenerative disorders. In addition to an overview of the results, we briefly outline the methods applied to the concept of resting-state functional connectivity.

While there are many different neurodegenerative disorders cumulatively affecting a substantial number of patients, for most of them studies on resting-state fMRI are lacking. Plentiful amounts of papers are available for Alzheimer's disease (AD) and Parkinson's disease (PD), but only few works being available for the less common neurodegenerative diseases. This allows some conclusions on the potential of resting-state fMRI acting as a biomarker for the aforementioned two diseases, but only tentative statements for the others.

For AD, the literature contains a relatively strong consensus regarding an impairment of the connectivity of the default mode network compared to healthy individuals. However, for AD there is no considerable documentation on how that alteration develops longitudinally with the progression of the disease. For PD, the available research points towards alterations of connectivity mainly in limbic and motor related regions and networks, but drawing conclusions for PD has to be done with caution due to a relative heterogeneity of the disease. For rare neurodegenerative diseases, no clear conclusions can be drawn due to the few published results. Nevertheless, summarising available data points towards characteristic connectivity alterations in Huntington's disease, frontotemporal dementia, dementia with Lewy bodies, multiple systems atrophy and the spinocerebellar ataxias.

Overall at this point in time, the data on AD are most promising towards the eventual use of resting-state fMRI as an imaging biomarker, although there remain issues such as reproducibility of results and a lack of data demonstrating longitudinal changes. Improved methods providing more precise classifications as well as resting-state network changes that are sensitive to disease progression or therapeutic intervention are highly desirable, before routine clinical use could eventually become a reality.

## Highlights

• Review of literature on resting-state fMRI connectivity in neurodegeneration.
• In diagnosing dementias resting-state fMRI bears potential to act as a biomarker.
• Most neurodegenerative diseases show distinct altered networks across studies.
• More studies and protocol harmonization will foster clinical resting-state fMRI.

## 1 Introduction

A biomarker is a usually indirect measure that accurately and reproducibly allows for an objective classification of a biological or pathogenic process or a pharmacological response (Strimbu and Tavel, 2011). There are also biomarker-surrogates, which have the same aims as a regular biomarker, but are an even less direct measure that is oftentimes easier to obtain that a *true* biomarker.

Resting-state fMRI connectivity can be seen as biomarker-surrogate, as while it does not directly capture neuronal processes and their connectivity it allows for insight in such characteristics. In general biomarkers should not only be able to identify the presence of e.g., a disease, but also should allow for tracking progression, severity and, importantly, treatment effects. This requirement holds particularly true for clinical trials studying neurodegenerative diseases, as their (usually) long courses complicate monitoring of clinical end-points and the often very long pre-clinical phases of these diseases hinder it entirely. As there is more and more evidence that interventions in neurodegenerative disorders need to be applied in very early or even pre-symptomatic phases of the respective disorder(s), monitoring of disease progression based on clinical features becomes well-nigh impossible, thus merely enforcing the need for reliable and easy-to-track biomarkers in this field. The usual measures of quality of an instrument, namely objectivity, reliability and validity apply to biomarkers as well (Strimbu and Tavel, 2011). In addition, the biomarker in question should be measurable easily and preferably non-invasively, should not require the cooperation of the patient (such as complying in highly demanding cognitive tasks, seeing that we are dealing with disorders of the brain) and should be widely available. Therefore, resting-state functional magnetic resonance imaging (fMRI) seems like an attractive choice, as it fulfils many of these requirements and available evidence so far points to it potentially being suitable for application as biomarker (Pievani et al., 2014).

Resting-state describes a task-free situation that is additionally characterised by very low levels of sensory stimulation. The concept itself is far from new and has been applied in neuroscience for a long time, although it may not always having been called explicitly *resting-state* (Snyder and Raichle, 2012). Not too long after the introduction of fMRI and the blood oxygen level dependant (BOLD) contrast (Ogawa et al., 1990), the combination of fMRI and the resting-state was used to assess connectivity of the brain (Biswal et al., 1995). Since then it has grown to become a popular and commonly used method in neuroscience and has of course also been applied within research on neurodegenerative diseases.

The question we here need to address when evaluating imaging (bio)markers is, if resting-state fMRI enables (i) to detect disease-specific changes compared to controls, (ii) these alterations are sensitive to disease progression and responsive to therapeutic intervention, and (iii) finally are reproducible and thus reliable.

**Fig. 1:**
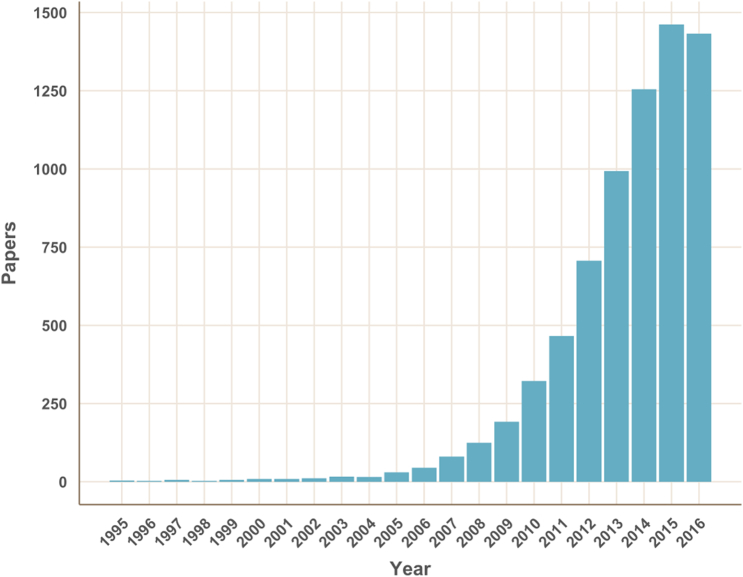
Amount of resting-state fMRI articles. Shown is the amount of results returned on PubMed for the query “resting-state fMRI” limited to each year from 1995 to 2016. It is can be seen easily that the amount of papers on that topic started to increase rather fast during recent years.

In principle, resting-state data can be gathered with functional methods different than fMRI, such as electroencephalography (EEG), magnetic encephalography (MEG) (van Diessen et al., 2015) and functional near infrared spectroscopy (fNRIS) (Niu and He, 2014). Due to the fact that it is now widely available, the cost manageable, the method being non-invasive and the spatial resolution of imaging very high, fMRI is by far the most common method used to collect resting-state datasets and the number of published papers employing it rose fast during recent years and remains high (Fig. 1).

In contrast to task-based approaches it could be argued that resting-state measurements provide a more neutral setting, as they do not elicit specific task-based activation. The neutrality of the resting-state condition comes with certain challenges, though. Besides numerous sources of error that might confound the data (Murphy et al., 2013), there is also considerable sensitivity of the measurement against the specific implementation of the resting condition such as eyes being closed, open or fixated (Patriat et al., 2013). Wandering of the mind should also be considered a source of variation of the data gathered (Mason et al., 2007), but that variation might be averaged out given a certain amount of data.

To characterise connectivity as measured with resting-state fMRI a wide variety of methods is available. An approach that is rather common is to correlate time series of brain regions (Biswal et al., 1995; Shehzad et al., 2009) and regard positive correlations as connectivity, while negative correlations (also called *anti-correlations*) have an unclear role. Similarly, common is the usage of independent component analysis (ICA) to identify brain networks. It aims to identify components of a data set by reducing statistical dependence between them, thus delineating data from different sources (Comon, 1994). It has been shown that the components identified by data correspond very closely to regions typically activated by task-based fMRI (Smith et al., 2009), are consistently measurable across healthy subjects (Damoiseaux et al., 2006) and show good test-retest reliability (Zuo et al., 2010). Further techniques to characterise connectivity based on resting-state fMRI include graph-theoretical approaches (Wang et al., 2010); Granger causality (Seth et al., 2015), as well as short-distance measures such as amplitudes of low-frequency fluctuations (ALFF) (Yang et al., 2007; Zang et al., 2007) and regional homogeneity (ReHo) (Zang et al., 2004). Of these measures, Granger causality is a measure of effective connectivity, while ALFF characterises features of individual regions. The here described methods are only a subset of techniques available for analysis of resting-state fMRI data and thus it is not surprising that integration of results is not without difficulties. Cole (2010) describes available methods and open questions regarding them in greater detail.

**Fig. 2:**
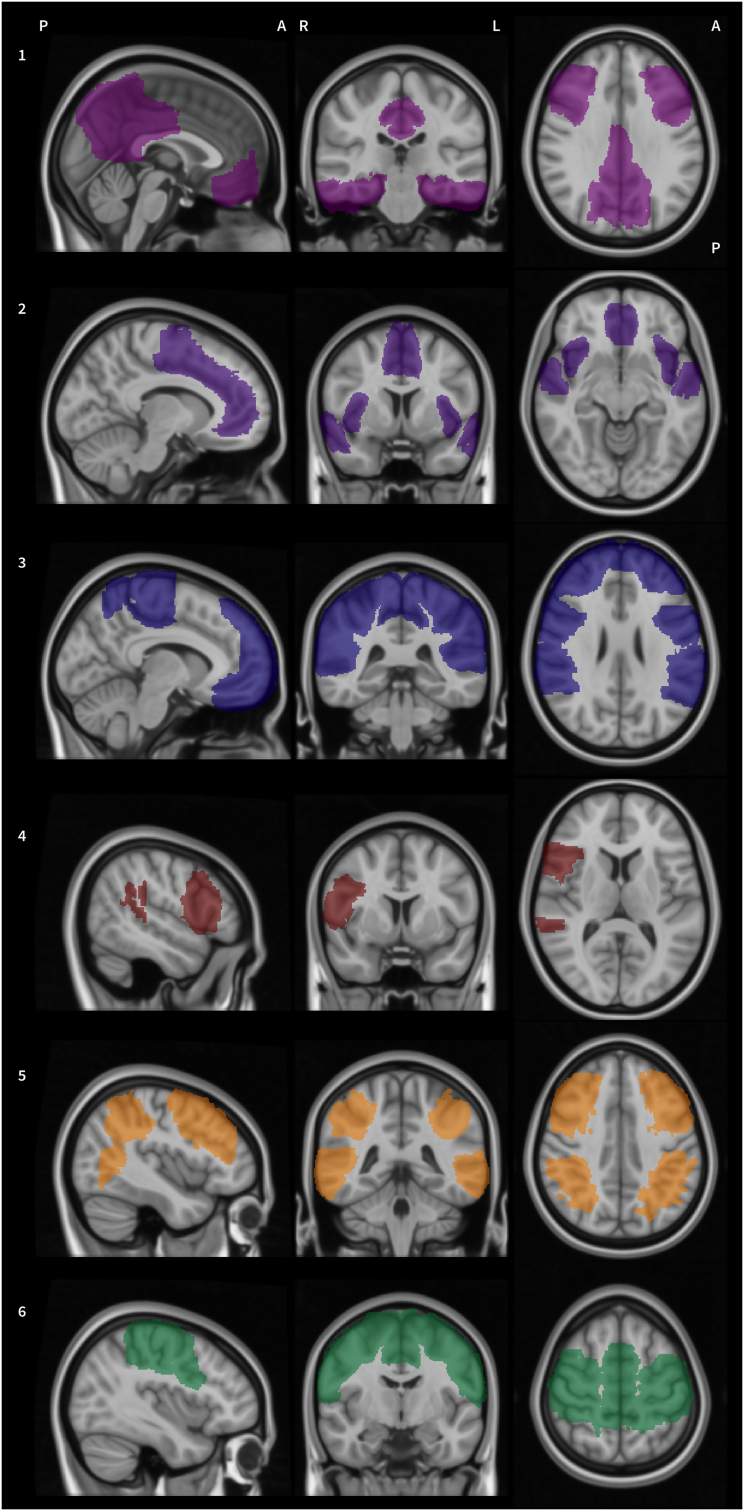
Visualisation of resting-state networks. Shown are approximations of 1) the default mode network; 2) the salience network; 3) the frontoparietal networks; 4) the ventral attention network; 5) the dorsal attention network and 6) the sensorimotor network. Regions involved in the networks were selected form the atlases provided with the FSL Eyes software (FSLEyes version 0.15.0, © FMRIB Centre, Oxford UK, https://fsl.fmrib.ox.ac.uk/). Images are superimposed onto the Colin-27 brain (Copyright (C) 1993–2009 Louis Collins, McConnell Brain Imaging Centre, Montreal Neurological Institute, McGill University).

Despite the large amount of analysis methods and difficulties in summarising results, a set of resting state networks in the brain have been identified and replicated many times in the literature. These networks are sets of brain regions that are interconnected serving a specific purpose. The most prominent example here is likely the *default mode network* encompassing the posterior cingulate, precuneus, inferior parietal cortex, orbitofrontal cortex, medial prefrontal cortex, ventral anterior cingulate, left dorsolateral prefrontal cortex, left parahippocampus, inferior temporal cortex, nucleus accumbens and the midbrain (Greicius et al., 2003; Raichle et al., 2001). It is believed to provide a baseline state of the brain that represents self-reference, emotional processing, memory as well as spontaneous cognition and aspects of consciousness (Raichle, 2015). Further networks include the *frontoparietal networks* – associated with numerous aspects of cognition and language processing (Smith et al., 2009; Zuo et al., 2010); the *sensorimotor network* relevant for motor execution and somatosensory components (Biswal et al., 1995; Smith et al., 2009); the two dorsal and ventral *attention networks* with the former associated with voluntary orientation and the latter linked to detection of salient targets (Corbetta and Shulman, 2002; Fox et al., 2006). Finally, the *salience network* is associated with the identification of relevant targets from the inputs the brain receives (Downar et al., 2000; Seeley et al., 2007), while the *executive control network's* main function is directing attention on such targets (Seeley et al., 2007). The location of these networks is visualised in Fig. 2.

If resting-state fMRI measurements could achieve similar quality in clinical practice, this would allow for rather fast and non-invasive diagnosis and tracking of progression in neurodegenerative disease and possibly beyond. Earlier reviews on this matter provide promising results and first evidence for potentially disease-specific patterns in connectivity alterations (Pievani et al., 2014). However as research is constantly expanding and methods are being developed, it should be re-assessed whether more light could be shed on these disease-specific patterns. Thus, we summarise the current state of the literature on functional resting-state-based results in neurodegenerative disorders in this review. The overall aim is to answer the question whether resting-state based data can serve as a non-invasive biomarker to diagnose and differentiate diseases.

## 2 Methods

Searches on PubMed (https://www.ncbi.nlm.nih.gov/pubmed/) were conducted for the following neurodegenerative diseases: Alzheimer's Disease (AD); Parkinson's Disease (PD); Huntington's Disease (HD); Multiple System Atrophy (MSA); Dementia with Lewy Bodies (DLB); Frontotemporal Lobar Degeneration (FTD); Amyotrophic Lateral Sclerosis (ALS); Creutzfeld-Jacob-Disease (CJD); Friedreich Ataxia (FRDA); and the Spinocerebellar Ataxias (SCA). The query “resting state” followed either by the name of the disease or a unique part of the name (such as *Alzheimer* for Alzheimer's disease) was used, the same was repeated with the search term “functional connectivity”. In addition, a small amount of papers was gathered from the reference sections of the papers collected using the PubMed searches. Studies were limited by time to include papers published up to the end of 2016. No limitations regarding journals for example based on impact factor were formulated. The search strategy was not explicitly limited to resting-state fMRI papers, but also included different methods. However, as the literature is dominated by resting-state fMRI, this review was subsequently limited to this method only, as we think that due to fundamental differences in acquiring data reviews focusing on one method at a time are for now able to provide more valuable insight.

**Fig. 3:**
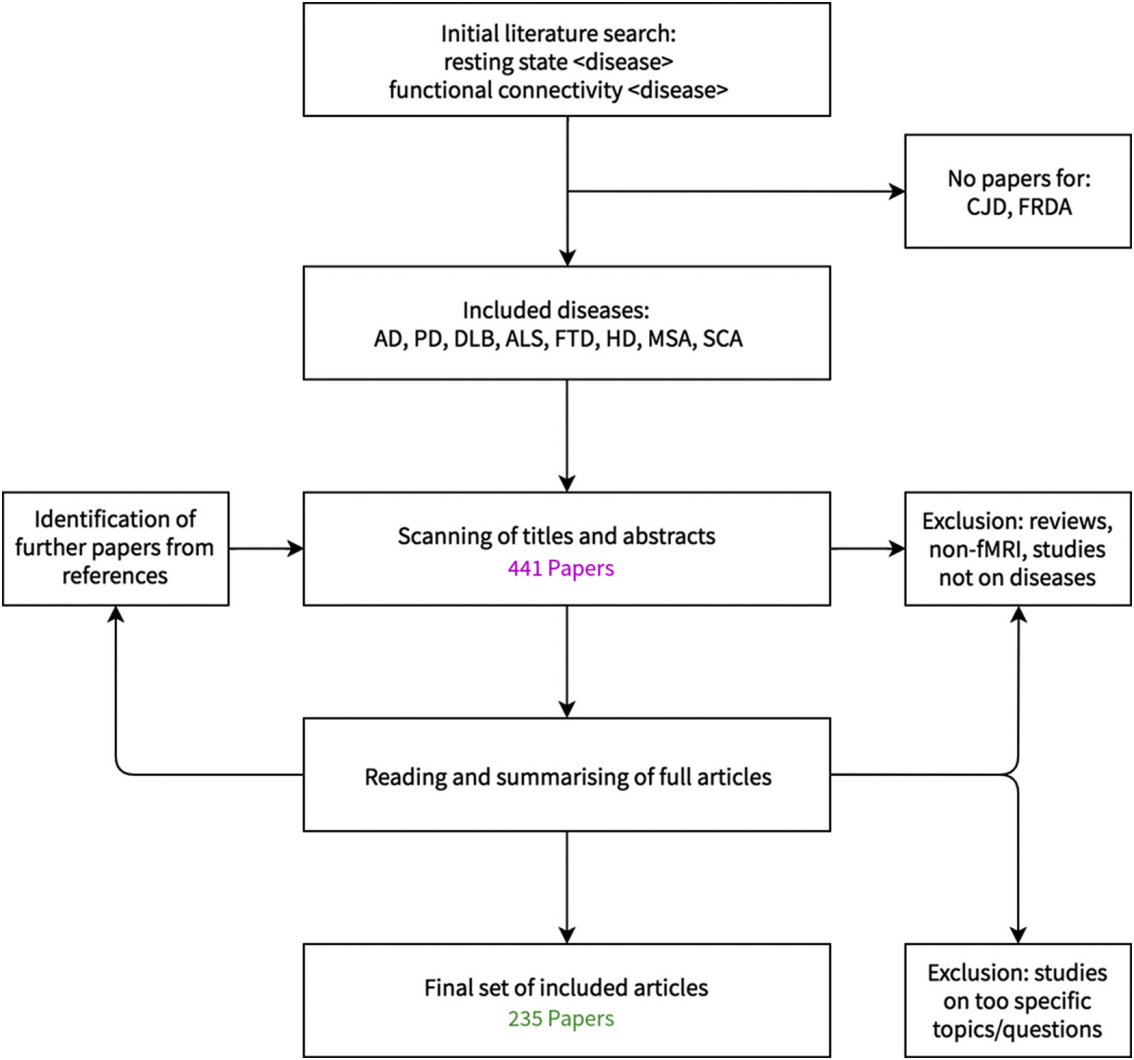
Schematic of the literature selection process. Illustrated is how papers were gathered and selected for potential inclusion into this review. An initial set of 437 papers was reduced to 231 papers on resting-state fMRI in neurodegenerative diseases contributing to the present review. Further referenced papers on methods, epidemiology and additional topics not directly communicating results of resting-state fMRI studies were not taken into account for this visualisation. Abbreviations: AD, Alzheimer's Disease; ALS, Amyotrophic Lateral Sclerosis; CJD, Creutzfeld-Jacob-Disease; DLB, Dementia with Lewy Bodies; FTD, Frontotemporal Dementia; FRDA, Friedreich's Ataxia; HD: Huntington's Disease; MSA, Multiple-Systems-Atrophy; PD, Parkinson's Disease; SCA, Spinocerebellar Ataxia.

As for some diseases no results were returned, the final list of diseases included in this review is as follows: AD, PD, HD, MSA, DLB, FTD, ALS and the SCAs. It should be noted that for these diseases, a substantial amount of literature is only available for AD and PD (95 and 68 papers included here), while for the other diseases the amount of literature is limited (up to 18 papers in this review), probably reflecting both their relative frequencies as well as funding policies. If a disease is further classified into subtypes, these are also respected in this review following the individual papers. We aimed to provide a general and broad overview of the resting-state fMRI literature for the neurodegenerative diseases, leading to the exclusion of papers dealing with very specific or narrow aspects of a disease or using either uncommon or novel methods for analysis. While exploration of (very) specific aspects of a disease and the development of novel methods are very important drivers of the scientific process, the lack of context to embed the results into made some of those studies unfortunately unsuitable for the aim of this review. A schematic overview of the literature selection process is given in Fig. 3. A key point to consider in any review is the nature of the publication bias, i.e. the tendency that studies that do not report results passing a certain threshold of statistical significance, are likely to never be published (Easterbrook et al., 1991). Especially for meta-analytic approaches this is a serious concern, but also for a review aiming to summarise the state of knowledge this essentially leads to the omission of findings and thus introduces a bias. While we did not account for potential bias, this should nonetheless be kept in mind when putting the summary provided in this paper into context.

A major challenge when summarising the state of the literature was the wide variety of used methods in the literature. This was especially a concern when trying to integrate findings from studies employing whole-brain approaches such as correlative analyses with regional measures like regional homogeneity measures (see below for more details on methods). Therefore, in some cases where regions of the brain affected by resting-state alterations were consistent between regions, but details on the findings were not (e.g., decreased versus increased connectivity, especially across methods), we fall back to describing this as a region, set of regions or network having altered connectivity or resting-state properties.

## 3 Evidence on resting-state fMRI in neurodegenerative diseases

**Fig. 4:**
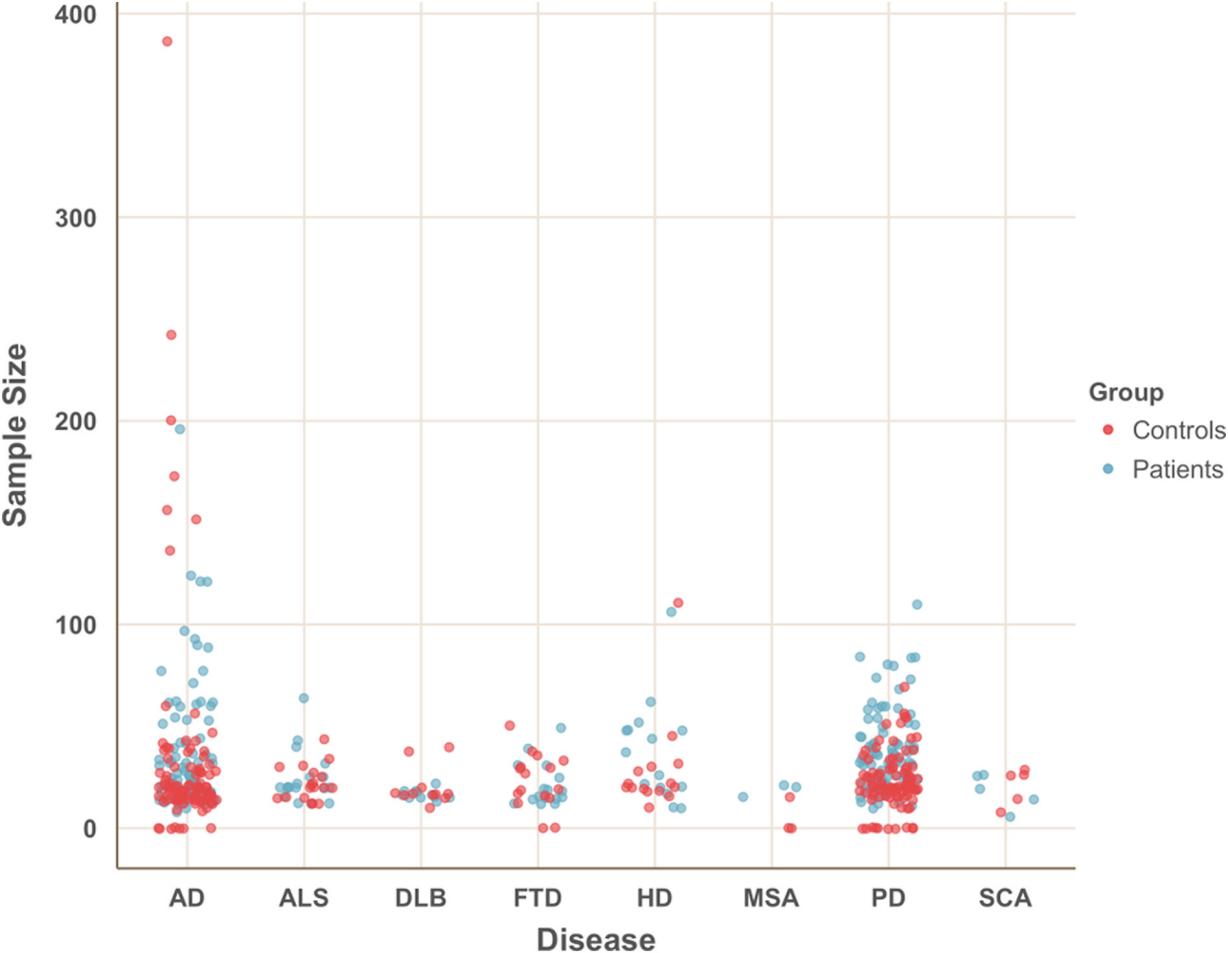
Sample sizes in papers on resting-state fMRI in neurodegeneration. Blue points show included sample sizes for patient groups, red points represent control subjects. Points are jittered to illustrate the distribution of sample sizes across papers for each disease.

Below, the state of the resting-state fMRI literature for the above-mentioned set of neurodegenerative diseases is summarised. A visualisation of sample sizes of patients or persons affected by factors associated with the eventual onset of a disease is given in Fig. 4. It should be noted that differences in sample size alone can lead to considerable differences of results and thus it might be problematic that sample sizes vary this much between studies.

### 3.1 Alzheimer's disease

AD is an age-associated disease, the most common form of dementia and the most common neurodegenerative disease. In persons aged 60 years and older, all dementias have a prevalence of about 4% and a yearly incidence of 7.5 per 1000 persons, with up to 70% of these cases being AD (Ferri et al., 2005). Its core symptom is cognitive decline most notably affecting numerous domains of memory. As society is aging and life expectancies are rising, AD poses a major threat and challenge, which is reflected in the amount of published research on it. For this review, we included 95 papers on resting-state functional connectivity in AD, including 3274 control subjects and 3406 patients of AD and MCI as well as asymptomatic persons at risk for eventual onset of AD, such as Apo ε4 carriers. These numbers are likely an overestimation due the re-use of datasets and use of data form databases like ADNI.

The term *prodromal AD* for the stage of the disease usually presenting with mild cognitive impairment of amnestic type (aMCI), but confirmed by liquor-based or amyloid imaging biomarkers as being due to AD has been introduced (Dubois et al., 2010). Many of the studies discussed below included an MCI or aMCI group, reportedly representing prodromal stages of AD. As this cannot be determined with certainty though, the terminology used by the individual papers is kept below.

#### 3.1.1 Hippocampal and default-mode network connectivity

Findings from resting-state fMRI in AD have been rather consistent. Early works on connectivity in AD have focused on the hippocampus, as this region is affected early and severely by the disease (Braak and Braak, 1991). It was found that in AD the hippocampus displays less connectivity than in healthy individuals to a broad spectrum of cortical and subcortical regions (Allen et al., 2007; Greicius et al., 2004; Li et al., 2002). This finding has been replicated in more recent works (Das et al., 2015; Kenny et al., 2012; Sohn et al., 2014; Tahmasian et al., 2015). With emerging interest in altered connectivity measured with resting-state focus shifted more towards broader networks, wherein the default mode network has seen a considerable amount of attention. While there is some variation in the precise regions reported as being affected by altered connectivity, there are some regions that are commonly mentioned as having altered connectivity in AD. These regions include the precuneus, posterior cingulate cortex and the prefrontal cortex, as nodes of the default mode network (Agosta et al., 2012; Balthazar et al., 2014b; Balthazar et al., 2014a; Binnewijzend et al., 2012; Brier et al., 2012; Damoiseaux et al., 2012; Filippi et al., 2013; Fleisher et al., 2009; Franciotti et al., 2013; Gili et al., 2011; Goveas et al., 2011; Griffanti et al., 2015; Hafkemeijer et al., 2015; He et al., 2007; Liu et al., 2014; Liu et al., 2012a; Miao et al., 2011; Qi et al., 2010; Schwindt et al., 2013; Sorg et al., 2007; Wang et al., 2006a, Wang et al., 2006b, Wang et al., 2007; Wang et al., 2011; Weiler et al., 2014; Wu et al., 2011b; Xi et al., 2012; Xi et al., 2013; Zhang et al., 2010; Zhang et al., 2012; Zhou et al., 2015). In fact, it is well established now that connectivity of the default mode network in impaired in AD. Further works also considered subdivisions of the default mode network. Based on the division into anterior and posterior default mode network, it was found that connectivity reductions in the default mode network are mainly found in the posterior default mode network (Koch et al., 2015), but with altered connectivity to the anterior default mode network (Jones et al., 2012; Song et al., 2013).

Despite a large amount of literature detailing characteristic alterations of default mode network connectivity in AD, there is a small number of papers reporting no such changes (Gour et al., 2011; Lowther et al., 2014; Soldner et al., 2012; Wang et al., 2013b). A reason for these findings that contradict the vast majority of the literature on resting state functional MRI connectivity in AD is likely the inclusion of patients with a high level of cognitive functioning or in particularly early stages of the disease.

#### 3.1.2 Networks beyond the default mode

Further networks have of course also been in the focus of research. For the anterior temporal network, an increase of functional connectivity has been found in early AD defined by a Clinical Dementia Rating (CDR) score of 1 (with 0 meaning cognitively normal and 3 meaning severe dementia), suggesting a compensatory mechanism to the onset of cognitive decline and increasingly altered cerebral connectivity (Gour et al., 2011). Contrary to that, a decrease of functional connectivity has been found for the dorsal attention network (but not its ventral counterpart) (Li et al., 2012a). For the salience network increased connectivity has been reported (Balthazar et al., 2014b; Zhou et al., 2010), but with contradicting evidence also being available (Filippi et al., 2013). Data is similarly inconclusive for the executive control network with some studies reporting no changes in AD (Gour et al., 2011) and others increased connectivity (Filippi et al., 2013; Weiler et al., 2014). Connectivity alterations characterised as increased connectivity in working-memory related networks were also found in AD (Filippi et al., 2013). Overall changes in connections between larger networks in AD have been reported as altered (Filippi et al., 2013; Li et al., 2013; Song et al., 2013).

Connectivity of specific subcortical regions and their networks such as the caudate nucleus has also been studied with the result that the caudate nucleus displays reduced connectivity to regions such as the posterior cingulate, cuneus and precuneus (Kenny et al., 2013). Similar results were found connectivity of the thalamus (Cai et al., 2015; Kenny et al., 2013), amygdala (Wang et al., 2016; Yao et al., 2013), marginal division (Zhang et al., 2014b) and locus coeruleus (Jacobs et al., 2015).

#### 3.1.3 Risk factors, genetics and early-onset

Some degree of alteration of resting-state functional connectivity is present in carriers of genetic mutations relevant to the eventual onset of AD, especially in carriers of at least one Apo ε4 allele, but also genetic mutations relevant for autosomal-dominant AD, and in subjects with a family history positive for AD. It is important to stress that the alterations can already be found in young adults carrying relevant genetic mutations, suggesting some potential for early identification of AD (Adriaanse et al., 2014; Brier et al., 2014b; Chhatwal et al., 2013; Dennis et al., 2011; Drzezga et al., 2011; Filippini et al., 2009; Li et al., 2014; Li et al., 2017; Machulda et al., 2011; Matura et al., 2014; Sala-Llonch et al., 2013; Sheline et al., 2010; Wang et al., 2015; Wang et al., 2012a). This shows that in congruence with the long pre-clinical stage of AD (Hulette et al., 1998; Sperling et al., 2014) functional alterations take place long before symptoms of the disease display.

While Alzheimer's generally is a relatively common disease, early onset AD is rather rare (Campion et al., 1999), and it is usually one of the genetic variants of AD. In comparison to sporadic AD, despite its genetic associations, autosomal-dominant forms of AD differ in clinical findings and are associated with a wide array of genetic factors (Bateman et al., 2011; Van Cauwenberghe et al., 2016). The term *early onset AD* includes AD with an onset of clinical symptoms before the age of 65. Only few studies researched whether there are distinct functional connectivity patterns therein. While in typical AD mainly the default mode network is affected by decreased connectivity and further networks show a mixed pattern, in early-onset AD it was reported that compared to similar-aged healthy individuals, connectivity is reduced over a broad set of regions (Adriaanse et al., 2014). Comparing typical and early-onset AD directly, the early onset group shows less connectivity in the auditory, sensorimotor, executive control, language, visual and dorsolateral prefrontal networks (Adriaanse et al., 2014; Gour et al., 2014; Lehmann et al., 2015). An opposite pattern has been shown for the antero-temporal medial network (Gour et al., 2014), and results for the default mode network are mixed (Adriaanse et al., 2014; Gour et al., 2014). Similar to what has been found for typical AD, an increase of connectivity for the anterior default mode network seems also to be present in early-onset AD (Lehmann et al., 2015).

#### 3.1.4 Graph theoretical approaches

There have been some studies employing graph theoretical measures to assess alteration of brain networks in AD. Here it was found that the degree centrality parameter is reduced in AD (Guo et al., 2016) and further works have shown a reduced clustering coefficient (Brier et al., 2014a; Chen et al., 2013; Sun et al., 2014; Supekar et al., 2008; Toussaint et al., 2014). Additionally, networks in AD were commonly found to have a loss of edges, especially of long-distance (Sanz-Arigita et al., 2010; Wang et al., 2013a; Zhao et al., 2012). Such properties have also been communicated for the small-worldness of the network in AD, with a negative correlation between small-worldness and disease severity (Brier et al., 2014a; Sun et al., 2014; Supekar et al., 2008; Toussaint et al., 2014). Similar overall network changes characterised by graph theoretical measures have also been found to be already present in Apo ε4 mutation carriers (Wang et al., 2015) and with increasing age (Toussaint et al., 2014). Interestingly, there is also a set of investigations that found an increase of the clustering coefficient in AD compared to healthy subjects (Liu et al., 2012b; Xiang et al., 2013; Zhao et al., 2012). More light on this seemingly contrary circumstance is shed by one paper in which a non-linear alteration of several graph theoretical measures by disease severity was revealed (Kim et al., 2015).

#### 3.1.5 Longitudinal changes

Considering the relatively large amount of works on resting-state functional connectivity in AD, there is only a rather small amount of works assessing long-term changes in this modality. A focus of the few longitudinal studies that have been conducted are patients that convert from a MCI stage to clinically fully manifest AD. Here, it was found that compared to the MCI stage, strength of connections between regions decreases, affecting both positive and negative correlations (i.e. both trend towards zero) (Bai et al., 2011; Hahn et al., 2013). Some differences between patients of stable (i.e. non-converting during the assessed time frame) MCI and those progressing to AD are supposedly already present before the conversion takes place (Esposito et al., 2013b). Other works have not found a difference between patients of MCI remaining in the same state and those that convert to manifest AD, though (Binnewijzend et al., 2012). One study did only administer connectivity changes over time in patients that were already suffering from manifest AD at baseline and found that overall strength of connectivity declined over time (Damoiseaux et al., 2012). Although it seems likely that connectivity further declines with progressing disease severity, there is virtually no data from highly affected (CDR ≥ 2) patients to support such an assumption, probably owing to the difficulty of obtaining informed consent from and measuring such severely ill patients. Overall, more studies on longitudinal changes of resting-state networks in AD are needed to further understand how neurodegeneration and network changes are related and to determine whether resting-state fMRI based methods could qualify as biomarker.

#### 3.1.6 Treatment effects

With a consistent finding of strongly altered brain networks in the resting-state in AD, of course the question is raised if and how these alterations can be counteracted upon by treatments. There has been some research on the effect of especially Donepezil on resting-state networks in AD (sample size between 8 and 18 CE patients). These have found that with no differences in study groups at baseline, the application of Donepezil leads to an increase in previously reduced connectivity. This was also reported to be correlated with behavioural scores, such as the Mini Mental State Exam (Goveas et al., 2011; Griffanti et al., 2016; Li et al., 2012b; Solé-Padullés et al., 2013). It should be noted that the extent of changes associated with Donepezil vary widely and more research is required to enhance understanding of this issue. There has been one additional study using an unspecified cholinesterase inhibitor (Wang et al., 2014a) in Apo ε4 carriers compared to non-carriers. Here, an increase in connectivity was also found after treatment compared to baseline. There have been additional studies on non-pharmacological interventions such as meditation (Wells et al., 2013) and acupuncture (Liang et al., 2014; Wang et al., 2012b, Wang et al., 2014b). While alterations of connectivity after treatment were found in all of these studies, the clinical validity of the treatment remains questionable here (Kaptchuk et al., 2000).

#### 3.1.7 Classification accuracy and conclusions

Considering that resting-state functional alterations are well documented and interest in AD remains high due to its relevance to society, the question remains whether resting-state functional measures can help in diagnosis of AD, ideally before the onset of symptoms. In fact, algorithms to classify subjects into either healthy subjects or patients of AD and/or MCI have been applied on the basis of resting-state functional data. These approaches had varying success with moderate to satisfactory performance (Balthazar et al., 2014a; Challis et al., 2015; Chen et al., 2011; Khazaee et al., 2015; Koch et al., 2012; Ni et al., 2016; Supekar et al., 2008; Wang et al., 2006a; Wang et al., 2013c; Wang et al., 2011; Zhou et al., 2010) reporting sensitivity in classification of patients against healthy controls of 72%–100% and specificity of 70%–95%. One study reported 100% classification accuracy in a small sample using SVM, but based on selection of discriminative features and without testing model performance in another data set, so caution is advised in interpreting these results (Khazaee et al., 2015). Especially the results of Koch et al. (2012) illustrate the need for combination of different data sources to achieve high sensitivity and specificity of classification, which is critical for the potential use of resting-state data as imaging biomarker. It needs to be kept in mind that the studies summarised above employed diverse analysis techniques so that at this point the suitability of resting-state fMRI for biomarker use remains unclear. In general, however, other measures such as grey matter volume perform better in classification of AD against healthy individuals (Schouten et al., 2016).

**Fig. 5:**
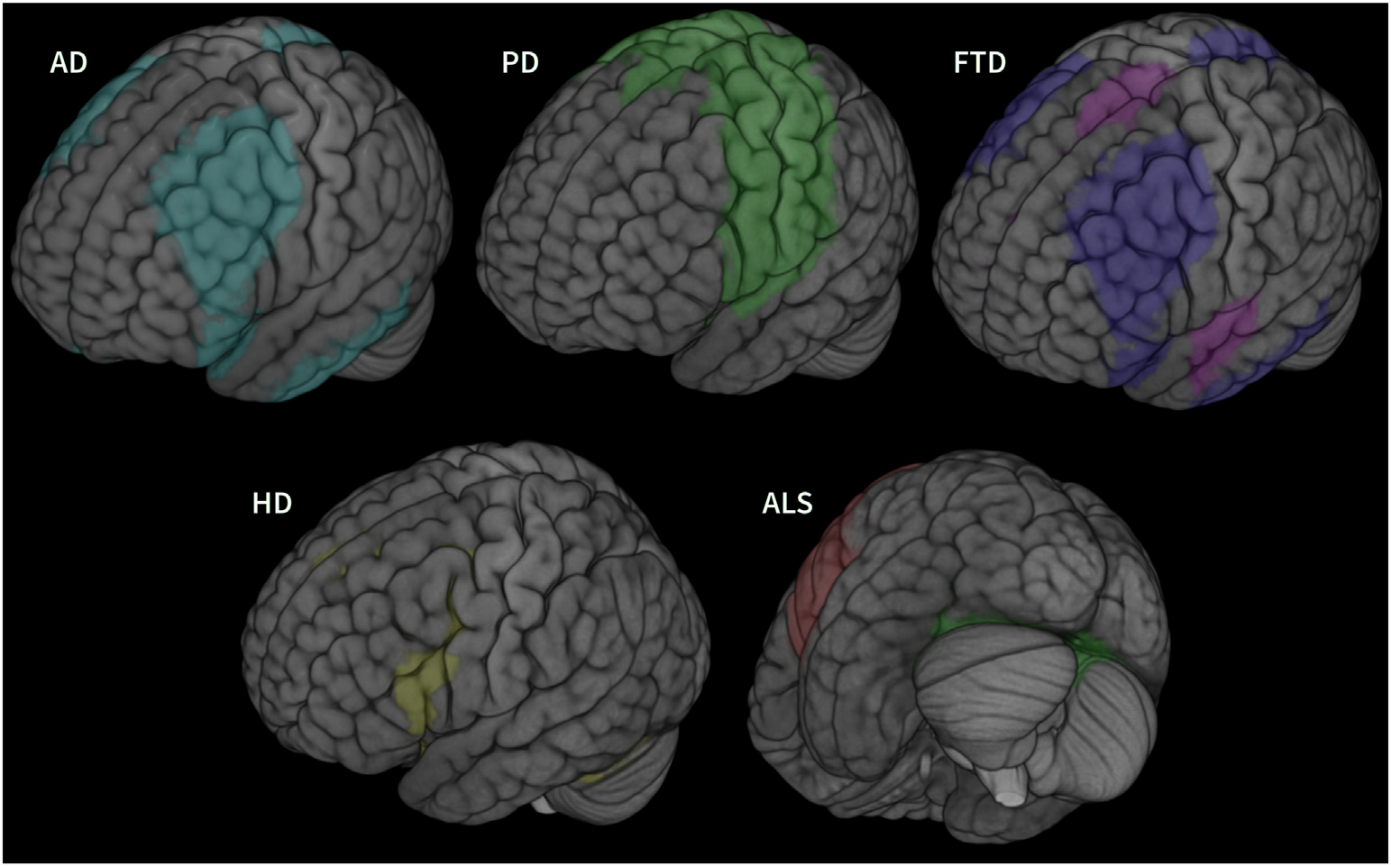
Schematic overview of the affected networks in AD, PD, FTD, HD and PD. The mainly affected regions for each disease are visualised on the cortical surface. For AD the default mode network, for PD the sensorimotor network and the basal ganglia, for FTD the default mode network (blue) and the salience network (pink), for HD motor and visual regions as well as the basal ganglia and for ALS the sensorimotor network (red) and visual areas (green). Due to few available studies and inconclusive evidence no visualisations for DLB, MSA and the SCAs are given. Regions were selected form the atlases provided with the FSL Eyes software (https://fsl.fmrib.ox.ac.uk/) and the AAL atlas (Tzourio-Mazoyer et al., 2002). Images are mapped onto a three dimensional rendering (created with MRIcroGL software (http://www.mccauslandcenter.sc.edu/mricrogl/home)) of the Colin-27 brain (Copyright (C) 1993–2009 Louis Collins, McConnell Brain Imaging Centre, Montreal Neurological Institute, McGill University).

In conclusion research on resting-state fMRI based connectivity and networks in AD has revealed characteristic patterns and alterations, especially regarding the default mode network (see Fig. 5 for a visualisation). These findings allow for a relatively satisfactory degree of discrimination of patients and healthy elderly. More focus is needed for networks beyond the default mode, on longitudinal development of resting state functional connectivity and how network properties integrate with various clinical variables, as there are not many publications investigating these topics yet. Compared to other networks, the default mode network has a special role, as it is not directly coupled to a specific function. Overall, the present evidence points towards rs-fMRI being able to identify AD patients, at first likely in addition to other markers such as measures of grey matter volume, later on possibly as a main criterion, becoming a reality in the not too distant future. Disease tracking based on a progressively increasing disintegration of networks could be a viable option, too. Research should now focus on identifying characteristic patterns of connectivity in AD that do not only involve the default mode network and simultaneously work at improving classifiers distinguishing patients from healthy individuals.

### 3.2 Parkinson's disease

Parkinson's disease (PD) is the second most common neurodegenerative disorder with a prevalence of 1.8 in 100 in persons aged 65 and above (de Rijk et al., 2000). It is mainly characterised by motor symptoms, but cognitive impairment and dementia are also commonly described. Further features include vegetative symptoms such as incontinence, gastrointestinal disturbances and hypotension, psychiatric symptoms such as apathy, visual hallucinations, depression and pain, and finally disturbances of deglutition, often the final and limiting set of symptoms. As it will be further outlined below, the disease is relatively heterogeneous with many described subgroups characterised by different symptoms, making conclusions for the overall disease more difficult. Nevertheless, the amount of literature on resting-state functional connectivity in PD is generally satisfactory and some valid conclusions can be drawn from it. In this review 68 papers were included covering 2492 patients with PD and 1685 healthy control subjects. Again, due to a presumptive re-use of data and data from repositories, these numbers are likely an overestimation.

#### 3.2.1 Connectivity of sensorimotor areas, the basal ganglia and associated regions

In PD connectivity alterations affecting motor regions including the supplementary motor area, the premotor area and the primary motor cortex, as well as the cerebellum were consistently found; this also includes the sensorimotor network as a whole entity. Further commonly reported changes involve the basal ganglia and their associated regions, the thalamus as well as the mesolimbic area (Agosta et al., 2014; Bell et al., 2015; Campbell et al., 2015; Canu et al., 2015; Esposito et al., 2013a; Hacker et al., 2012; Ham et al., 2015; Helmich et al., 2010; Kwak et al., 2012; Li et al., 2016; Luo et al., 2015b; Luo et al., 2014b; Rieckmann et al., 2015; Rolinski et al., 2015; Sharman et al., 2013; Simioni et al., 2016; Skidmore et al., 2013b; Szewczyk-Krolikowski et al., 2014; Wu et al., 2011a, Wu et al., 2015; Wu et al., 2009a; Wu et al., 2009b; Yu et al., 2013). Baik et al. (2014) found connectivity in PD to be modulated by striatal dopamine levels. An overall clear direction of connectivity alterations (i.e. increase or decrease of connectivity) does not seem to emerge from the literature.

For the striatum, a trade-off between connectivity of its anterior and posterior portions was reported – with an increase in connectivity of the anterior striatum, while connectivity of the posterior striatum declines and vice versa, in a way mirroring neuropathology (Helmich et al., 2010). Data are further available for the substantia nigra with impaired connectivity to the putamen in the left hemisphere and increased connectivity to cuneus and precuneus in the right hemisphere (Ellmore et al., 2013). For the subthalamic nucleus – another key region in PD – it was found that connectivity is increased to a wide range of regions including the primary motor cortex, supplementary motor area, premotor area and somatosensory cortex (Baudrexel et al., 2011; Fernández-Seara et al., 2015; Kurani et al., 2015). Another investigation of the connectivity of this region reported that only negative connectivity of it is altered in PD (Mathys et al., 2016). Connectivity of the amygdala was also the subject of research on connectivity in PD. Here, the amygdala displays impaired connectivity to frontal, occipital and cerebellar locations (Hu et al., 2015a).

A study specifically aiming to delineate cerebellar connectivity in PD found an association between connectivity alterations with increasing atrophy. Motor parts of the cerebellum display disrupted connectivity to the default mode, sensorimotor and dorsal attention networks and increased connectivity to the frontoparietal network and the cognitive portions have reduced connectivity to the sensorimotor network (O'Callaghan et al., 2016).

The default mode network has also been subject of research in PD. Unlike in AD, no clear patterns regarding the default mode network are present in the literature. Some papers report either no (Helmich et al., 2010; Krajcovicova et al., 2012) or only few (Canu et al., 2015; Disbrow et al., 2014; Kurani et al., 2015; Lucas-Jiménez et al., 2016; Tessitore et al., 2012) alterations of default mode network connectivity. But a study aiming to distinguish patients of PD from healthy subjects found rather substantial alterations of the default mode network in disease (Chen et al., 2015b), and one report found an additional connection not present in healthy subjects from the default mode network to right central executive network (Putcha et al., 2015). Generally, increased default mode network connectivity was also found in some papers (Campbell et al., 2015; Gorges et al., 2015). Widespread alterations of networks in PD, within and between networks, that include, but are not limited to the default mode network, have been reported as well (Madhyastha et al., 2015; Onu et al., 2015). An investigation of the executive control network did not yield any differences in PD compared to healthy adults (Disbrow et al., 2014), but another study found impaired connectivity between the right central executive network and the salience network in PD (Putcha et al., 2015). Disruptions of a network associated with vocalisation have been reported in one study (New et al., 2015). Finally, some studies pointed out that functional connectivity in PD seems to be partially related to olfactory performance (Su et al., 2015; Sunwoo et al., 2015).

Using graph theoretical measures on resting-state data in PD, it was reported that network efficiency is decreased, with a higher clustering coefficient and characteristic path length (Göttlich et al., 2013; Wei et al., 2014). Results on early-stage patients under treatment, are generally compatible with global efficiency being reduced (Sang et al., 2015). Regions affected by these changes overlap with those elaborated on above and include motor regions such as the primary motor cortex, premotor cortex, supplementary motor cortex, also the somatosensory cortex, the basal ganglia and the cerebellum (Ghasemi and Mahloojifar, 2013; Göttlich et al., 2013; Koshimori et al., 2016; Sang et al., 2015; Wei et al., 2014). A study implementing functional connectivity density mapping found decreased short range connectivity mainly in cerebellar and visual regions and decreased long range connectivity in superior and middle frontal gyri. Increased short and long range connectivity were found for the posterior cingulate and precuneus (Zhang et al., 2015a).

#### 3.2.2 Akinesia, tremor and freezing of gait

Only few studies explicitly compared both main types of PD. In tremor-dominant PD compared to akinetic-rigid PD it was reported that ALFF is higher in cerebellar locations and the putamen, but lower in temporal and superior parietal areas (Chen et al., 2015a). Comparing ReHo between both types, higher values were found for tremor-dominant PD in the putamen, inferior parietal lobe, area S1, superior temporal and inferior frontal gyrus; with lower values for the thalamus, posterior cingulate, precentral gyrus, superior frontal gyrus and cerebellar locations (Zhang et al., 2015b). Compared to healthy subjects impaired cerebellar connectivity was found again in tremor-dominant PD, and disrupted precentral connectivity for akinetic-rigid PD (Hu et al., 2015c). One further study found differences in default mode network connectivity in akinetic-rigid PD compared to tremor-dominant PD, but also a lack of differences between patients of tremor-dominant PD compared to healthy subjects (Karunanayaka et al., 2016).

The term *freezing of gait* describes the episodic inability to initiate or maintain movements and is often associated with advanced PD. There are some studies that have explicitly researched how freezing of gait reflects in resting state functional connectivity in PD. Some differences between PD with freezing of gait and PD without it have been found. This includes alterations of connectivity affecting the supplementary motor area and caudate compared to PD without freezing of gait, but the available literature does not point towards a typical pattern yet, but rather contains heterogeneous results (Canu et al., 2015; Fling et al., 2014; Lenka et al., 2016; Vervoort et al., 2016).

#### 3.2.3 Parkinson's disease with depression

Regarding PD with depression compared to PD without depression it was found that connectivity differs from regular PD in the way that in frontal regions an impairment is present, encompassing the prefrontal cortex, superior frontal gyrus, orbitofrontal gyrus and extending to the anterior cingulate, but in parietal and cerebellar areas connectivity is enhanced in PD with depression (Lou et al., 2015; Luo et al., 2014a; Wen et al., 2013). Altered frontal connectivity in similar regions, but with different directions (increased) were reported elsewhere (Sheng et al., 2014). The amygdala is an additional important region in PD with depression, here altered connectivity was to a wide range of regions including the frontal and temporal cortices, the putamen and cerebellum (Hu et al., 2015a; Huang et al., 2015). One study found an association between depressive symptoms in PD and alterations of frequency bands in the subgenual cingulate (Song et al., 2015). There is also a result of no whole-brain changes in connectivity in PD with depression, but nevertheless correlation between severity of symptoms of depression and PD with localised connectivity measures (Skidmore et al., 2013a).

#### 3.2.4 Parkinson's disease with dementia

Dementia is prevalent in PD and can impact functional connectivity. Connectivity in this condition has not been researched well, but the findings that are available suggest that connectivity of the default mode network and of the extrastriate visual network is impaired compared to both PD and healthy subjects (Gorges et al., 2015; Rektorova et al., 2012). Alterations of the default mode network seem to vary with disease duration (Shin et al., 2015). In addition, evidence regarding the direction of alteration is unclear, as increased connectivity was reported also (Baggio et al., 2015a). Further reports show disrupted connectivity in frontal and parietal networks in PD with dementia (Amboni et al., 2014; Borroni et al., 2015), as well as in the dorsal attention network (Baggio et al., 2015a).

#### 3.2.5 PD with rapid eye movement sleep behaviour disorder

Rapid eye movement (REM) sleep behaviour disorder (RBD) is considered to be a prodromal stage of α-synucleinopathies and thus associated with the eventual onset of PD. The available literature on resting-state measures in RBD indicates an alteration of connectivity between the left putamen and substantia nigra (Ellmore et al., 2013). As a recent review pointed out, while there is an overlap in affected regions to PD, changes are not as strong in RBD. However, due to the limited amount of available literature, no conclusions can be made regarding an eventual progression from RBD to PD (Heller et al., 2016). One study researching specific connectivity alterations in PD with RBD compared to PD without RBD found decreased ALFF in the primary motor cortex and premotor cortex when RBD was present in addition to PD (Li et al., 2016).

#### 3.2.6 Further features in PD

A considerable amount of further symptoms in PD has explicitly been focused on in the resting-state fMRI literature, but only very few published studies are available for these. These are summarised briefly. Comparing early onset PD to late onset PD using ReHo, differences were found in the right putamen and left superior frontal gyrus (Sheng et al., 2016). In PD patients suffering from pain resulting from the disease, a disconnection between right nucleus accumbens and left hippocampus as well as altered orbitofrontal and cerebellar fractional ALFF were revealed compared to PD patients without pain (Polli et al., 2016). An investigation of connectivity in PD with regular falls found increased connectivity in the central executive network in patients reporting falls compared to those who did not report falls (Rosenberg-Katz et al., 2015). Patients of apathetic PD were found to display decreased functional connectivity affecting the limbic system compared to non-apathetic patients (Baggio et al., 2015b). The presence of hallucinations in PD was associated with higher default mode network activity and disruptions in inter-network connectivity between ventral and dorsal attention network (Franciotti et al., 2015; Shine et al., 2014; Yao et al., 2014, Yao et al., 2015). Comparing patients of PD with hyposmia to those without, a wide array of connectivity alterations was found affecting frontal and temporal regions, including, but not limited to, those associated with olfactory processing (Su et al., 2015). For PD with fatigue reduced sensorimotor network connectivity and increased default mode network connectivity compared to PD without fatigue was found (Tessitore et al., 2016). One article assessing connectivity in PD with impulse control disorders mainly found a disconnection between left anterior putamen and left inferior and anterior cingulate gyri (Carriere et al., 2015). Additionally two studies are available that did assess influence of genetics associated with PD that suggest that connectivity alterations are present before onset of disease when genetic risk factors are present (Makovac et al., 2016; Vilas et al., 2016).

#### 3.2.7 Clinical associations

A common finding is that connectivity measures mainly in, but not limited to, motor regions are associated with symptom severity as operationalised by the motor scale of the Unified Parkinson's Disease Rating Scale (UPDRS) (Baudrexel et al., 2011; Luo et al., 2015a; Putcha et al., 2015; Wu et al., 2011a; Wu et al., 2009b). There is some conflicting evidence though, as some works found mainly associations between connectivity measures and cognition, but not motor assessments (Disbrow et al., 2014; Olde Dubbelink et al., 2014). An association between graph characteristics and cognitive performance was reported in one study (Lebedev et al., 2014). Overall, there is no consensus between studies which specific connections correlate with behavioural measures.

#### 3.2.8 Treatment effects

The effect of pharmacological treatment on resting-state connectivity in PD has been researched rather well. Several studies report that when comparing patients in the on-state (i.e. medication is currently administered) with the off-state, connectivity measures change towards the state found in healthy subjects (Agosta et al., 2014; Bell et al., 2015; Esposito et al., 2013a; Kwak et al., 2010, Kwak et al., 2012; Wu et al., 2009b). Increased connectivity from the posterior cingulate to the precuneus and posterior cingulate, but no overall default mode network differences were found under dopaminergic medication (Krajcovicova et al., 2012).

A study on functional connectivity after deep brain stimulation of the subthalamic nucleus has found that deep brain stimulation improves cortico-striatal and thalamo-cortical connections and reduces connectivity from the target regions of the stimulation – the subthalamic nucleus (Kahan et al., 2014). Another paper investigating the effects of deep brain stimulation of the subthalamic nucleus in PD found altered connectivity affecting the brainstem and cerebellum after insertion of electrodes, but in the off state; and increased premotor connectivity correlating negatively with UPDRS score when electrodes were activated (Holiga et al., 2015).

A study on automatic mechanical peripheral stimulation demonstrated an impact on sensorimotor and striatal connectivity (Quattrocchi et al., 2015).

#### 3.2.9 Longitudinal changes and disease stages

There are a few assessments on changes in resting-state fMRI connectivity in the long term in PD, but these are of essential value for the development of a biomarker. A study on changes over three years found that connectivity constantly declined in PD, mainly in sensorimotor regions and the occipital lobe (Manza et al., 2016; Olde Dubbelink et al., 2014). Analysing fALFF, alterations mainly in temporal and occipital locations were found elsewhere (Hu et al., 2015b). A systematic cross-sectional assessment of connectivity in PD by Hoehn & Yahr stage found that alterations are the most pronounced in stage 2 and affect precuneus/posterior cingulate, right lateral parietal lobe, left inferior occipital gyrus and left lingual gyrus. Connectivity between occipital and temporal regions was revealed to decrease with Hoehn & Yahr stage (Luo et al., 2015a).

#### 3.2.10 Classification approaches and conclusions

There have been approaches to distinguish patients of PD from healthy subjects. These have had overall moderate to satisfactory success (Chen et al., 2015b; Fernández-Seara et al., 2015; Long et al., 2012; Onu et al., 2015; Skidmore et al., 2013b; Wu et al., 2015; Zhang et al., 2014a), although improvements need to be made to apply these in routine use. Reported sensitivity and specificity of classification when trying to distinguish patients of PD and healthy individuals is usually around 90%–95%. Particularly good classification results were found based on basal ganglia networks (Rolinski et al., 2015; Szewczyk-Krolikowski et al., 2014). Generally, classification results were better when measures such as volumes of white matter and grey matter were also included in the classification process.

Drawing overall conclusions is difficult despite a satisfactory amount of literature due to the heterogeneity of the PD phenotype. In addition, as many studies do not explicitly differentiate their cohorts based on the presence of certain features, overall results might be weakened. For example, one paper showed an absence of effects when not accounting for presence of cognitive impairment (Baggio et al., 2015a). It seems plausible that similar effects could be true for other subtypes and symptoms of the disease as well, so that this should be addressed more in future research. Regions associated with the sensorimotor network and the basal ganglia seem to be commonly affected by altered connectivity in PD (see Fig. 5), but more work is needed to further characterise these changes and to understand how they vary between phenotypes of PD.

### 3.3 Dementia with Lewy Bodies (DLB)

DLB is the second most common neurodegenerative form of dementia after AD, but it is a rare disease with its prevalence in persons of 65 years and older having been reported to be about 7 in 1000 (McKeith et al., 2004). In DLB, attention, visuo-spatial cognitive functioning and domains associated with subcortical areas are affected most prominently, while memory is not affected as severely. Further symptoms include predominantly visual hallucinations, fluctuating cognition, psychiatric symptoms, as well as parkinsonism and additional motor symptoms which need to be present within a year from the onset of cognitive symptoms, thus defining the border to dementia resulting from PD (Geser et al., 2005; McKeith et al., 2004). For this review we included eleven papers including 223 healthy controls and 181 patients with DLB.

The default mode network has been assessed in several studies on resting-state functional connectivity in DLB. Default mode network connectivity was reported to be unchanged in DLB with fluid cognition in one study (Franciotti et al., 2013). In contrast there is evidence that parts of the default mode network are affected by changed connectivity (Galvin et al., 2011a; Kenny et al., 2012; Lowther et al., 2014; Peraza et al., 2015a) with overlap in reported regions towards the cerebellum and visual processing areas. Findings of patients with DLB displaying altered connectivity in visual areas and networks have also been reported in the literature (Peraza et al., 2014; Sourty et al., 2016), suggesting that changes in this domain might be characteristic for the disease.

Additional networks that have received attention are the right and left fronto-parietal networks. These have also been reported to display changes in connectivity in patients with DLB compared to healthy individuals (Peraza et al., 2015a). One investigation found that the left fronto-parietal networks does not only display reduced connectivity, but that this also correlates with cognitive fluctuations (Peraza et al., 2014).

Further connectivity changes that have been reported in DLB affect subcortico-cortical connectivity (Kenny et al., 2013), the sensorimotor network (Peraza et al., 2016), the salience and executive control networks (Lowther et al., 2014) as well as the fusiform gyrus and pons (Borroni et al., 2015), but regarding these findings the literature is lacking in number.

Finally, there is one report employing a whole-brain graph theory based approach. Here, alterations of connectivity are characterised by increased global efficiency, increased nodal clustering and decreased characteristic path length. Looking at local alterations, parietal and posterior temporal areas have been found to have decreased nodal degree in patients of DLB compared to healthy subjects (Peraza et al., 2015b).

A large part of the body of literature available on DLB also includes a comparison against AD, but no consistent differences seem to emerge from such analyses (Franciotti et al., 2013; Galvin et al., 2011b; Kenny et al., 2012, Kenny et al., 2013; Lowther et al., 2014; Peraza et al., 2016; Peraza et al., 2015b). When comparing DLB against PD with dementia – both diseases involve Lewy bodies – the localisation of regional homogeneity alterations differs between the diseases (Borroni et al., 2015), but seed-based analysis suggests no difference between both diseases (Peraza et al., 2015b). This is concerning for potential diagnostic use of resting-state data, but comparisons on large datasets might yield more reliable results.

In summary, the literature on resting-state functional connectivity in DLB is rather limited and inconclusive at this point. Connectivity alterations seem to be widespread, but so far there is no consensus regarding their localisation and extent. In particular, differentiation from AD or PD seems to pose severe problems as of now. Consequently, more research is necessary to characterise DLB in terms of functional network connectivity.

### 3.4 Multiple System Atrophy

Multiple System Atrophy (MSA) is a rare neurodegenerative disease characterised by autonomic failure, parkinsonian symptoms and/or cerebellar features. Depending on the dominant symptoms a parkinsonian (MSA-P) and a cerebellar type (MSA-C) are commonly described (Fanciulli and Wenning, 2015; Stefanova and Wenning, 2016). MSA is a rare disease, having a prevalence of about 3–4.5:100,000 (Bjornsdottir et al., 2013; Schrag et al., 1999).

The amount of literature on MSA and resting-state functional connectivity is rather small with only three published papers of which one included both subtypes, and the other two only patients of MSA-P. Overall these three papers include 15 control subjects and 56 MSA (45 MSA-P, 11 MSA-C) patients. Clear-cut conclusions are thus very difficult to make, but nevertheless we shortly summarise the state of the literature.

There is some overlap in regions reported with altered resting-state connectivity in MSA including the frontal lobe, or more specifically motor related areas in superior and middle gyri, as well as the prefrontal cortex (Franciotti et al., 2015; You et al., 2011). The ReHo analysis by You et al. (2011) further pointed out that right frontal areas have increased regional homogeneity, with left areas having decreased regional homogeneity. Additionally the parietal lobule (left: increase, right: decrease) and posterior cingulate (decrease) were reported to be affected. This seems to fit well into data of decreased frontoparietal and parietocingulate connectivity (Franciotti et al., 2015). While the work by Franciotti et al. (2015) did only include MSA-P patients, the study by You et al. (2011) included both subtypes, but pooled them for the comparison against healthy controls. A further analysis between both types showed that regional homogeneity differed in the right primary somatosensory and motor cortices.

A further study investigated the effects of a rTMS intervention on resting-state connectivity in MSA-P (Chou et al., 2015). It shows that modulation of resting-state functional networks in MSA is possible using rTMS, but does not provide insight of differences in resting-state networks between MSA patients and healthy subjects.

In summary, the literature on resting-state functional connectivity in MSA does not allow for conclusions, as the amount of literature on that matter is far too small. There is some overlap between regions with changed roles in resting-state reported, but no further literature is available to verify these first results.

### 3.5 Amyotrophic Lateral Sclerosis

Amyotrophic Lateral Sclerosis (ALS) is a rare neurodegenerative disease with a yearly incidence of about two cases per 100,000 people (Logroscino et al., 2008, Logroscino et al., 2010). Its most important symptoms affect upper and lower motor neurons, but the clinical phenotypes vary greatly (Brooks et al., 2000). Motor symptoms affect the limbs; include difficulties of speech, swallowing and respiration; as well as overall weakness. The disease progresses relatively fast, leading to death in about 50% of patients within 30 months after onset (Kiernan et al., 2011). While there are some genetically determined forms, the majority of cases is sporadic in nature. This review includes 18 papers on resting-state fMRI in ALS incorporating a total of 395 healthy controls and 442 patients of primarily sporadic ALS, which is limited, but allows for some conclusions to be drawn.

In ALS, decreased connectivity of the orbitofrontal cortex and increased connectivity of the precuneus was found, both as part of the default mode network (Agosta et al., 2013a). There is recent evidence for unchanged connectivity in the default mode and the sensorimotor networks in ALS compared to healthy subjects (Chenji et al., 2016) and findings of impaired default mode network connectivity (Mohammadi et al., 2009; Tedeschi et al., 2012).

The sensorimotor network has repeatedly been shown to be impaired in ALS. This mainly affects areas such as the premotor area, supplementary motor area, primary motor cortex, the somatosensory cortex and the somatosensory association cortex (Fang et al., 2016; Mohammadi et al., 2009; Tedeschi et al., 2012; Zhou et al., 2014). Further motor-related connectivity changes, affecting additional regions were reported elsewhere (Trojsi et al., 2015; Zhou et al., 2013).

In addition to sensorimotor network differences, a recent study reported differences between patients of ALS and healthy individuals in a network incorporating the precuneus, cingulate and middle frontal gyrus (Menke et al., 2016). Focusing explicitly on the occipital cortex, impaired connectivity between the right occipital cortex and bilateral precuneus in ALS has further been reported (Zhang et al., 2016). The frontoparietal network was reported to be affected by decreased connectivity of the inferior frontal lobe and increased connectivity of the angular gyrus and parietal cortex in general (Agosta et al., 2013a).

Zhu et al. (2015) found increased ALFF in patients of ALS compared to healthy controls in the right parahippocampus, left inferior temporal gyrus, left anterior cingulate, right superior frontal gyrus and left middle occipital gyrus. Increased ALFF was further reported to be found in the right inferior frontal gyrus and left middle frontal gyrus, and decreased ALFF right postcentral gyrus and bilateral fusiform and inferior occipital gyri (Luo et al., 2012). This is quite different from other resting-state fMRI based findings on ALS, but this is likely due to methodological differences, especially as ALFF is not a direct assessment of connectivity.

There are also studies employing graph theoretical measures to assess network changes related to ALS. An assessment of degree of centrality found numerous alterations in ALS. Increased degree of centrality measures were found for bilateral regions of the cerebellum, the occipital lobe, lingual gyrus, right orbitofrontal gyrus and left superior frontal gyrus, while decreased degree centrality was shown for the bilateral precuneus, further occipital areas including the right calcarine gyrus and left lingual gyrus, left middle temporal and Heschl gyri, left supramarginal gyrus, right insula, right inferior frontal gyrus and precentral gyrus, left postcentral and paracentral gyrus as well as the left cingulate (Zhou et al., 2016). Another report compared functional connectivity strength between patients of ALS and healthy subjects based on networks identified by graph theory measures. There, it was found that patients of ALS displayed increased functional connectivity strength in frontal areas, namely the right orbitofrontal cortex, bilateral medial superior frontal gyrus and right superior and middle frontal gyri (Ma et al., 2015).

In conclusion, while the literature on resting-state fMRI in ALS is lacking in number, it points towards possible characteristic patterns of changed connectivity in ALS. The sensorimotor network seems to be affected very pronouncedly in ALS and its alterations might therefore qualify as a biomarker. In fact, alterations of resting state networks in ALS have already been used together with machine-learning to differentiate between healthy subjects and patients of ALS (Fekete et al., 2013; Welsh et al., 2013) mainly relying on data from the default mode and sensorimotor network. While the reported classification accuracies are likely not high enough for routine clinical application, they are nevertheless promising. One study comparing ALS to behavioural variant FTD, two diseases belonging to a single spectrum of disorders mediated by in part the same genetic defects, pointed out similarities, but also differences affecting the sensorimotor and default mode networks, that might be of future diagnostic use (Trojsi et al., 2015). Further studies investigating networks integrating several imaging modalities, including resting-state fMRI, but also structural data such as tractography, may provide further information to identify patients of ALS (Douaud et al., 2011; Schmidt et al., 2014), but they are beyond the scope of this review. There is moreover evidence, mainly from ALFF and graph-theory based studies, that points towards an involvement of the visual system in ALS pathology, which needs to be investigated in future studies. Overall, the reported findings need further validation, though and most importantly longitudinal assessments to become a potentially viable tool to diagnose ALS as early as possible.

### 3.6 Frontotemporal dementia (FTD)

Frontotemporal dementia (FTD) is a rare disorder with an estimated incidence of about 3:100,000 (Onyike and Diehl-Schmid, 2013). At the same time it is also the third most common form of neurodegenerative dementia. FTD can be mainly divided into two different types on initial presentation: a behavioural variant (bvFTD) and a language variant including semantic dementia (seFTD) and progressive non-fluent aphasia, but all eventually develop into a global dementia (Gorno-Tempini et al., 2011; Neary et al., 1998; Rascovsky et al., 2011). Regarding resting-state functional MRI, the amount of available literature is rather limited, but nevertheless provides a relatively clear-cut pattern. For this review, we included 16 papers on functional connectivity in FTD, including a total of 371 control subjects and 341 patients of FTD variants and subjects at risk for eventual onset of FTD, such as carriers of mutations of the GRN gene associated with the eventual onset of FTD. It is important to note that most resting-state fMRI studies in FTD have focused on bvFTD, with only two studies including seFTD patients and one study investigating patients of phenocopy FTD (phFTD) – a variant of bvFTD (Davies et al., 2006).

A common finding of studies assessing networks is that connectivity of the salience network is impaired in FTD (Caminiti et al., 2015; Day, 2013; Farb et al., 2013; Filippi et al., 2013; Premi et al., 2013; Trojsi et al., 2015; Zhou et al., 2010). Furthermore, while not reported as often as previous results, this is commonly found together with increased default mode network connectivity (Farb et al., 2013; Meijboom et al., 2016; Rytty et al., 2013; Trojsi et al., 2015; Zhou et al., 2010), but connectivity reductions within the default mode network have also been reported (Farb et al., 2013; Filippi et al., 2013). Further research has demonstrated alterations of connectivity of further networks such as the executive control network, dorsal attention network, the auditory network (Hafkemeijer et al., 2015) and the frontoparietal and frontotemporal networks including the insular cortex (Farb et al., 2013; Rytty et al., 2013; Sedeño et al., 2016). While for each network more research is needed to confirm and clarify the findings, a commonly shared feature among them and also in studies not explicitly characterising functional networks (Agosta et al., 2013b; Jastorff et al., 2016), is that they include frontal regions.

Studies that also looked at associations between measures for disease severity in FTD and connectivity measures reported various associations between them (Agosta et al., 2013b; Caminiti et al., 2015; Day, 2013; Farb et al., 2013; Sedeño et al., 2016; Trojsi et al., 2015; Zhou et al., 2010) mainly showing a positive correlation between symptom severity and the degree of network alterations.

Where types of FTD different than bvFTD were measured, results point towards differences in connectivity between subtypes, but the extent seems limited. For phFTD less increased connectivity in the default mode network compared to bvFTD was shown, localised in the medial prefrontal cortex, lateral temporal cortex and inferior parietal cortex (Meijboom et al., 2016). Regarding seFTD, a smaller alteration of salience network connectivity compared to bvFTD was reported, with a further study showing differences especially in prefrontal and cingulate connectivity between seFTD and bvFTD (Day, 2013; Farb et al., 2013).

Three studies on resting-state fMRI in FTD investigated the role of genetic mutations relevant to the eventual onset of FTD pathology. This is especially true for the GRN mutation, but numerous other genetic mutations have been linked to the eventual onset of FTD (Bang et al., 2015). It was found that given the presence of these mutations, alterations of connectivity are already found in asymptomatic carriers. These alterations were mainly found in frontoinsular regions, i.e. in regions overlapping with those affected in manifest frontotemporal dementia (Dopper et al., 2014; Premi et al., 2014, Premi et al., 2016).

Comparing patients of bvFTD and AD directly, it could be shown that regarding the default mode network and the salience network opposite patterns seem to emerge between both diseases. The default mode network shows increased connectivity and the salience network appears to have disrupted connectivity in bvFTD with an opposite pattern being present in AD (Zhou et al., 2010). Elsewhere, additional inter-network as well as network-to-region alterations were reported comparing bvFTD and AD (Hafkemeijer et al., 2015).

In conclusion, alterations of functional connectivity in frontotemporal dementia are very promising as a stand-alone biomarker. Although in order to guide interventional studies, data on longitudinal changes due to the natural history of the disease are sorely needed. However, rs-fMRI might provide diagnostic capabilities, because it is a particular concern that cases of FTD are commonly misdiagnosed as AD (Jellinger, 2006). Given that resting-state functional alterations are fundamentally different and in case of the default mode network contrary than in AD (a direct comparison can be seen in Fig. 5), a resting-state functional assessment could be an important tool to support the diagnostic process.

### 3.7 Huntington's disease (HD)

HD is a rare neurodegenerative disorder with a prevalence about 5:100,000 (Walker, 2007), which is characterised by motor symptoms, cognitive impairment and psychiatric symptoms. It is a devastating disorder usually leading to death after a course of 10–15 years after manifestation. On conventional, i.e. structural, imaging, patients with HD show very specific patterns of cerebral atrophy, most notably affecting the striatum (Tabrizi et al., 2012).

The literature commonly differentiates between preHD individuals, who carry the gene mutation associated with HD, but do not show symptoms of the disease yet, and those individuals who are already affected by motorically manifest HD. Regarding functional connectivity changes, there are some longitudinal works, but none that systematically track the changes occurring on manifestation of HD. An issue regarding drawing conclusions from the literature on resting-state in HD is the broad methodological variety, making it relatively difficult to draw proper conclusions from the available literature. For this review 15 studies were included, encompassing 431 controls and 570 patients of both pre-manifest and manifest HD.

#### 3.7.1 Pre-manifest Huntington's disease

Decreases in connectivity are found the earliest from the left frontal, right parietal and both occipital cortices to the medial visual network as well as reduced connectivity from the bilateral cingulate (Dumas et al., 2013). A later study found presumptive compensatory network changes in the right hemisphere, but not in the left, and no changes in motor-related connectivity. The extent of compensatory connectivity was related with white and grey matter loss in the caudate and putamen adjusted by total intracranial volume (Klöppel et al., 2015). Impaired cortico-striatal connectivity between the caudate nucleus and lateral motor areas in preHD were reported by Unschuld et al. (2012). Investigations of connectivity of M1 in preHD found it to be disrupted (Poudel et al., 2014) and dependent on genetic disease burden measured by CAG repeat length (Koenig et al., 2014). Connectivity changes that increase with genetic disease burden are decreases in connectivity between M1 and contralateral motor and somatosensory cortices, bilateral cuneus and an increase with the posterior cingulate. Finally, there is also a report of no difference in connectivity between healthy and preHD individuals, though (Seibert et al., 2012).

Longitudinal studies did not find any changes in networks in preHD individuals over the course of one to three years (Odish et al., 2014; Seibert et al., 2012). Considering the reported association between disease load and network changes this seems contradictory, but it should be kept in mind that the preHD phase has to be regarded as the entire phase starting from conception until motor manifestation, which can take place at any age but usually peaks between the fifth and the sixth decade. It is assumed that there is a non-linear course of neuropathology (Walker, 2007).

Using graph theoretical analyses in preHD, a mostly intact network was reported so far, with the organization of network hubs decreasing (Gargouri et al., 2016; Harrington et al., 2015) mainly affecting sensorimotor and associative regions. Using a more detailed approach, classifying preHD subjects according to their disease progression it became apparent that the degree of network changes increases with nearing disease onset mainly manifesting as increased network efficiency, suggesting an increasing number of shortcuts between regions (Harrington et al., 2015). In conclusion, there is evidence for changes in functional connectivity changes specific to pre-manifest HD affecting motor-associated and visual regions as well as the striatum, but due to the small amount of studies with a wide range of employed measures, at this time no clear conclusions can be made.

#### 3.7.2 Manifest Huntington's disease

Generally, the alterations found in preHD are usually also present in manifest HD, but seem to be more pronounced and extended to further regions and networks. The medial visual network was shown to be affected in manifest HD with reduced connectivity to this network further spanning the orbitofrontal cortex, subcortical grey areas including the globus pallidus, putamen and thalamus. Additionally, the default-mode-network was affected by reduced connectivity from prefrontal, left parietal and bilateral temporal areas. Finally, the left supramarginal gyrus and both thalami displayed reduced activation to the executive control network (Dumas et al., 2013; Quarantelli et al., 2013). One study did not find the connectivity changes affecting the cingulate in preHD in manifest HD, though (Dumas et al., 2013), while alterations of cingulate connectivity to the default mode network were reported elsewhere (Quarantelli et al., 2013). There is also a report of a strongly affected dorsal attention network in manifest HD including right sensorimotor and left supramarginal, paracingulate, angular and superior frontal gyri (Poudel et al., 2014). In our own study, we found mainly increased connectivity in early HD, including subcortical regions such as the thalamus, caudate nucleus, putamen, but also the cerebellum, large parts of the frontal cortex including motor areas and mainly superior parietal regions (Werner et al., 2014). Included in this finding are the precuneus and the anterior cingulate as hubs of the default mode network. In addition, it was also reported that connectivity between distinct networks was reduced, supposedly indicating a loss of long-range functional coupling between networks. Both increases and decreases of connectivity were reported in one study (Wolf et al., 2014b), with early patients of HD displaying increased connectivity in motor and further frontal areas, as well as the caudate nucleus while parietal and temporal areas as well as the cingulate were found to display decreased connectivity. Further, altered visual system integrity was found, with decreased left fusiform gyrus and increased left cerebellar activity (Wolf et al., 2014a). Differential cerebellar connections in manifest HD as compared to healthy were found from cerebellar lobule VIIa to paracentral, lingual and inferior frontal areas (Wolf et al., 2015).

A single study measured ALFF in HD. Decreases were found in the bilateral precuneus, right posterior cingulate and right angular gyrus. Increases were reported for the left inferior and middle temporal gyrus, left fusiform gyrus, left superior frontal and middle orbitofrontal gyrus as well as the right inferior temporal gyrus (Liu et al., 2016).

There is also one report making use of graph theory measures in manifest HD (Gargouri et al., 2016). Extending upon the findings in preHD it was reported that the cortical network approached randomness over a two-year period, including a loss of sensorimotor and associative network hubs, and changes in numerous graph theory measures.

#### 3.7.3 Conclusions

The available literature demonstrates that HD has a significant impact on resting-state networks, which is already present in the pre-manifest stage of the disease, but there is a lack of studies demonstrating a longitudinal progression from preHD to manifest HD and also within both stages of HD in terms of resting-state functional connectivity. This is especially critical, as the long-term reports in preHD did not show any connectivity changes over time. While the regions reported to have altered connectivity in both preHD and manifest HD are overlapping to some extent, there is still a large amount of open questions due to conflicting findings. Future research should address at which the patterns observed in preHD turn into those more extensive network changes in manifest HD. Moreover, network changes in HD that can be measured across different methods should be pinpointed to a characteristic pattern of network changes in HD, although it can be argued that the overlap in areas associated with the visual system, motor-related regions and structures of the basal ganglia is large enough to already show a characteristic pattern. At the moment, the wide variety of measures and methods employed in the somewhat limited literature poses the main obstacle in identifying a valid and reliable resting-state fMRI biomarker in HD.

### 3.8 Spinocerebellar ataxia

The spinocerebellar ataxias (SCA) are a group of neurodegenerative diseases with autosomal-dominant inheritance. The main symptom is ataxia that can be accompanied by additional symptoms according to the SCA type. As the prevalence for all spinocerebellar ataxias is at most 5:100,000, they are all rare diseases (Ruano et al., 2014). Regarding resting-state fMRI a very small amount of literature is available, with one paper on SCA1, two papers on SCA2 and two papers on SCA7. In total these papers report on 103 healthy controls and 91 patients of SCA. As the SCAs are individual diseases with a very broad spectrum of clinical phenotypes, results for the individual subtypes cannot be generalised to all SCAs.

Regarding SCA1 connectivity of the cerebellum and the thalamus is altered in patients. This was characterised as a simplification of networks (Solodkin et al., 2011).

For SCA2 a decrease of functional connectivity was shown for several cortical networks, namely the default-mode-network, the executive control network and the right fronto-parietal network. Some increases in connectivity were also found, but only in the default mode network. A further investigation of mainly cerebellar connectivity revealed wide changes of connectivity involving this region. This was related to behavioural measures assessing disease severity (Cocozza et al., 2015; Hernandez-Castillo et al., 2015).

Finally, for SCA7 altered cerebellar connectivity was reported as well. This mainly includes a decrease of connectivity between the cerebellum and a wide set of frontal and motor-related regions. Also, it was reported that the synchrony between the cerebellum and the frontal lobe is impaired in SCA7. The amount of connectivity alterations correlates with CAG-repeat-extension and disease duration. Classification accuracy based on functional connectivity for SCA7 from healthy controls was relatively good (Hernandez-Castillo et al., 2013; Hernandez-Castillo et al., 2014).

Given the available literature on SCA types, no conclusions beyond the individual reports can be made and more research is required to gain a thorough understanding of alterations of cerebral connectivity in the SCA.

## 4 Conclusions

The core findings of this review in regards to summarising alterations of resting-state fMRI-based connectivity are: for AD, an impairment of connectivity of the default mode network is well established in the literature with heterogeneous evidence regarding further networks. In PD, alteration of motor and limbic connectivity seems to be a common pattern, but the heterogeneity of the disease calls for caution in interpreting these results. In DLB, connectivity alterations are widespread, but so far the literature does not suggest a common pattern. In MSA an involvement of motor and overall frontal regions seems to be common, but more research is necessary to validate these findings. For ALS, alterations of sensorimotor regions seem to be the main result from resting-state fMRI, with further evidence suggesting potential relevance of the default mode network and the visual system. A pattern of decreased default-mode connectivity and increased salience network connectivity appears to be characteristic for FTD. The literature available on HD suggests a pronounced alteration of the connectivity of sensorimotor regions and the basal ganglia – especially the striatum – with further evidence towards alterations of the connectivity of the visual system. Finally, for the SCAs cerebellar connectivity alterations seem to be present, but at this point in time this should not be interpreted as a common pattern given the small amount of literature and the very limited generalisability across the genotypes. The relative uncertainty outlined here already points towards resting-state fMRI being unfit for biomarker use at this point in time, but the key issues are discussed in detail below.

A major challenge regarding resting-state fMRI in neurodegeneration is the relative rarity of diseases, making researching them rather difficult. Multi-centric approaches may pose a solution to a certain degree here. As for multi-centric approaches, there is the issue of effects induced by different hardware used. While not extensively documented, it has been shown that different MRI scanners lead to different effects in various neuroimaging modalities, even if two devices of the same model are compared against each other (Grech-Sollars et al., 2015; Han et al., 2006; Takao et al., 2013). This might also be a source of noise between studies, as those are of course most often conducted using different scanners. Considering that there is substantial variation between results even for a disease like AD, where the amount of literature is rather large, it seems possible that only the most prominent patterns persist across studies and scanners, but more subtle results are not reproducible or, even worse, are the result of hardware effects. However, endeavours such as TRACK-HD (Tabrizi et al., 2009) show that using standardised protocols and rigorous adherence to them can yield substantial results across multiple centres in rare diseases when examining structural MRI data. It seems very plausible that this should also hold true for resting-state fMRI data. Nevertheless, at this point in time the issue of variability of results between different scanners appears to be an issue that needs to be addressed further before routine-use of resting-state fMRI as a biomarker could be an option. As the definition of a biomarker involves reliability, a lack of re-test reliability between hardware is a serious aspect. Without being able to delineate what is noise due to hardware and what is objectively measured signal, resting-state fMRI – and also different MRI modalities – cannot be used as a biomarker.

In regard to whether the above outlined findings could be leveraged to identify patients suffering from a particular disease, i.e. to use the resting-state fMRI-based alterations as diagnostic biomarker, there is not enough evidence to support this idea for any of the diseases. For most diseases there are no studies trying to differentiate patients and healthy controls as well as patients of similar diseases. Where such studies are available it is disappointing to see that at this point in time they do not report more successful rates of classification. From a research perspective correct classification reported in the literature so far are promising results, but for routine clinical use even higher values are needed. Especially critical here is that a classification approach also works across samples and studies and is not limited to only one small set of patients. Additionally, works so far have mostly been on distinguishing patients from healthy controls, but it is still unknown whether this also works for disease progression or on a large set of different diseases. Considering only imaging data, analysis of grey matter volumes seems to provide more insight into disease state than functional imaging. This is clearly illustrated by works systematically comparing different imaging modalities for patient classification, which points towards resting-state data not only performing worse in classification when used alone, it also does not provide a large enhancement when added to a classifier that incorporates different modalities of MRI data (Schouten et al., 2016). Possible solutions for this issue might be the application of more refined classification algorithms; especially the field of machine learning seems to bear considerable potential. Furthermore, inclusion of several characteristics at the same time to drive the classification might also lead to improved results. Finally, the eventual availability of faster fMRI sequences has also potential to leverage progress here, as with decreased lag between measurement time points, quality of data is improved.

Considering resting-state fMRI-based connectivity measurements as a biomarker with discriminative qualities only, practical applications could probably already be implemented at this point in time. This is true for cases where the diagnosis is uncertain or where it is known that diseases are often misdiagnosed, such as non-AD dementias being diagnosed as AD. The application of a short resting state scan and subsequent data analysis could lead to clarity already at this time.

One particular issue that becomes apparent when summarising the literature available is that there do not seem to be clear patterns indicative of disease progression in any of the included diseases. This is starkly illustrated in HD, where the conversion from preHD to manifest HD in terms of resting-state connectivity has not been described yet, despite clear differences in both disease states. However, this is an essential feature of a biomarker, which would perform rather poorly if it were unable to point out changes in severity of a disease. Some of the literature on AD is more promising with resting-state fMRI-based connectivity measures being altered in young mutation carriers (Dennis et al., 2011; Filippini et al., 2009), but there is no study yet employing a longitudinal tracking of alterations over an extended amount of time. Given that results in resting-state fMRI studies are usually calculated on a group-level it could very well be argued that while individual progression could be measured with resting-state fMRI, the variance between patients is too large for measuring progression on a group-level. Systematic longitudinal tracking of resting-state measures in individual patients could potentially provide further insight into this issue. One fundamental problem in regard to evaluating the potential of resting-state fMRI for use as biomarker is the plethora of analytical methods employed and, further aggravating the situation, the lack of standardised protocols for even data acquisition of neuronal and non-neuronal signals, for instructions to patients, and for dealing with noise (*pre-processing* of data). While the latter is typical for the entire field of fMRI research and does not seem to influence results in task-based fMRI too much, where an external behavioural standard is available, it poses a severe problem in resting-state fMRI, where results are entirely based on some sort of correlation of one measured source of signal with many others. Pre-processing rs-fMRI data has evolved into an art form on its own, with an entire field of research to accompany it. But also the multitude of statistical approaches that is finally applied to the purified data makes comparisons across studies extremely difficult, as results from one method cannot be compared well to results from a study employing another technique, even if the same terms are used for describing results such as *network strength* or *connectivity*. Great care has to be taken when comparing studies employing seed-based correlations with ICA-based methods; both of which are basically incomparable to studies measuring regional features such as ALFF or ReHo. Graph theoretical approaches are even further removed from the biological processes under review, as they describe the organisational features of a network on a meta-level and could (and are) as easily be applied to social or financial networks. To apply this situation to potential use of resting-state fMRI as biomarker, without a standardised analysis technique aimed at this use case, it will simply not be possible to put this idea into practice. Research should now try to delineate this issue with systematic comparisons of various analysis techniques under special consideration of disease identification, differentiation and longitudinal tracking.

Regarding the overall question whether there is potential for an imaging biomarker based on resting-state fMRI in neurodegenerative diseases it can be said that while there seems to be potential, at this point in time it seems unclear whether this will become a reality and we are much less optimistic in this regard than earlier works on this matter which did identify similar changes in resting-state fMRI based connectivity measures and came to the conclusion that this modality is promising for biomarker use (Pievani et al., 2011). The main issues at the moment are 1) for AD and PD, where a substantial amount of data is available, classification accuracy is not good enough for routine use; 2) for the less common diseases, the amount of literature available even prevents a theoretical foundation for a biomarker; 3) a lack of studies assessing resting-state measures longitudinally, thus obviating the use of fMRI as a monitoring tool for disease progression. Even more fundamentally, the lack of a precisely defined terminology and standardised protocols both mean that it is not possible right now to delineate which aspects of resting state activity or connectivity might be able to bridge these gaps on a group level, let alone on an individual level.

Future studies should aim to address these points, as unifying protocols for gathering and analysing data must be considered as the perhaps important step towards the development of an resting-state imaging biomarker in neurodegenerative diseases.

## Conflict of interest statement

The authors report no conflicts of interest.
